# Interpretation Bias in Breast Cancer Survivors Experiencing Fear of Cancer Recurrence

**DOI:** 10.3389/fpsyg.2021.682174

**Published:** 2021-11-18

**Authors:** Malwina Tuman, Kailey E. Roberts, Geoffrey Corner, Courtney Beard, Carol Fadalla, Taylor Coats, Elizabeth Slivjak, Elizabeth Schofield, Wendy G. Lichtenthal

**Affiliations:** ^1^Department of Psychiatry and Behavioral Sciences, Memorial Sloan Kettering Cancer Center, New York, NY, United States; ^2^Ferkauf Graduate School of Psychology, Yeshiva University, Bronx, NY, United States; ^3^Department of Psychology, University of Southern California, Los Angeles, CA, United States; ^4^McLean Hospital, Belmont, MA, United States; ^5^Harvard Medical School, Boston, MA, United States; ^6^Department of Psychology and Neuroscience, Boulder, CO, United States

**Keywords:** fear of cancer recurrence, interpretation bias, somatic symptoms, breast cancer survivors, mediation

## Abstract

**Introduction:** Fear of cancer recurrence (FCR) is a prevalent and persistent challenge that many cancer survivors endure. While the role of interpretation bias, a tendency to perceive ambiguous situations as threatening, has been established in the onset and maintenance of FCR, few studies have examined cancer-related interpretation bias specifically. Grounded in the cognitive formulation of FCR, the current study aimed to fill this gap by investigating the relationship between cancer-related interpretation bias, FCR, and somatic symptoms, and examining whether bias mediates the relationship between somatic symptoms and FCR.

**Materials and Methods:** This study used baseline data from a randomized controlled trial of a cognitive bias modification intervention. Breast cancer survivors (*n* = 110) provided demographic and medical background information as well as self-report measures of FCR and severity of somatic symptoms. A computer-based assessment of interpretation bias was used to measure cancer-related interpretation bias on several bias indices: percentage of cancer-related threat endorsement, and percentage of benign endorsement; mean reaction time (RT) for threat, and mean RT for benign endorsement.

**Results:** Higher threat endorsement was linked to higher Overall Fear and emerged as a mediator of the relationship between overall somatic symptoms and Overall Fear. We also found that older age was related to longer benign endorsement RT.

**Conclusion:** This study contributes understanding of factors related to cancer-related interpretation bias and provides evidence that bias may influence the relationship between somatic symptoms and FCR in cancer survivors.

## Introduction

Fear of cancer recurrence (FCR) is a prevalent problem in cancer survivors. With estimated rates ranging from 39 to 97%, cancer survivors have identified managing FCR as their top unmet need (Simard et al., 2013). Some degree of FCR is adaptive for managing medical follow-ups and motivating health-promoting behaviors (Simonelli et al., 2017), but excessive FCR can compromise quality of life through psychological distress, functional impairments (Simard and Savard, 2015), and maladaptive behaviors, including hypervigilance for symptoms of recurrence in the future.

Assessment of FCR has posed challenges. Although there exist over 30 instruments measuring FCR, many provide no psychometric data, and few offer clinical cut-off scores to identify those most in need of intervention (Smith et al., 2018). Assessment is further complicated by the multidimensional nature of FCR, which incorporates several factors including triggers activating FCR, the severity of intrusive thoughts surrounding FCR, psychological distress, coping strategies to manage FCR, functioning impairments, insight regarding the intensity of FCR, and reassurance behaviors (Simard and Savard, 2009). Despite a growing body of research on factors associated with FCR, few correlates have been identified as consistent and “strong” predictors (Humphris and Ozakinci, 2008). Thus, identifying common contributors underlying the etiology and maintenance of FCR, empirically validating their relationship with FCR, and identifying potential intervention targets remain research priorities (Lebel et al., 2017).

One known potent trigger of FCR is interpreting physical symptoms as potential indicators of cancer recurrence (Crist and Grunfeld, 2013; Hall et al., 2019). Cognitive formulations of illness representation suggest that if appraised as potential symptoms of recurrent disease, benign somatic experiences can elicit a fear response (Easterling and Leventhal, 1989; Lee-Jones et al., 1997; Fardell et al., 2016). This is consistent with cognitive theories of anxiety, which propose that biased information processing, such as interpreting ambiguous information as threatening can contribute to elevated anxiety (Ouimet et al., 2009; Chen et al., 2020). Interpretation bias involves a tendency to interpret external or internal information in a negative manner (see Hirsch et al., 2016 for a review) and has been implicated in health anxiety (Antognelli et al., 2020), pain (Heathcote et al., 2016), chronic fatigue syndrome (Hughes et al., 2016), cancer-related fear (Miles et al., 2009), distress (Lam et al., 2018), and FCR (Lichtenthal et al., 2017).

This study was grounded in a similar cognitive formulation specific to FCR and its antecedents (Lee-Jones et al., 1997). The cognitive formulation of FCR suggests that if external (e.g., follow-up oncology appointments) or internal cues (e.g., somatic symptoms) are appraised as potentially threating, corresponding negative cognitions can result in elevated FCR. Increased cancer fears can, in turn, lead to maladaptive behaviors (e.g., excessive body checking) and greater health anxiety, exacerbating the tendency to interpret environmental and internal cues as cancer-related (i.e., cancer-related interpretation bias) and ultimately perpetuating a cycle of maladaptive thoughts and behaviors and emotional distress.

Because interpretation bias has been linked to the development and exacerbation of impairing anxiety symptoms, a broad range of experimental paradigms have been developed to explore (Schoth and Liossi, 2017) and modify (Beard and Peckham, 2020) interpretation bias, including the Word Sentence Association Paradigm (WSAP; Beard and Amir, 2009; Gonsalves et al., 2019), a reliable and valid assessment of interpretation bias across a variety of populations. Our team utilized WSAP to assess changes in interpretation bias in a randomized clinical trial (RCT) of a cognitive bias modification (CBM) intervention to target FCR in breast cancer survivors.

While this intervention resulted in significant reduction in cancer-related interpretation bias and FCR-related health worries measured post-intervention and at a 3-month follow-up (Lichtenthal et al., 2017), the presumption that interpretation bias, FCR, and somatic symptoms were correlated at baseline was not established. Endorsement of a greater number of physical symptoms has been associated with greater FCR (Hall et al., 2019), but the mediating role of implicit cognitive processes in this relationship has not yet been investigated. Finally, although few correlates have emerged as consistent predictors of FCR, there is evidence that certain demographic and medical characteristics including disease stage, time since treatment completion, age, being a parent, and having racially/ethnically minoritized status may be linked to FCR (Crist and Grunfeld, 2013; Simard et al., 2013). However, the relationship between these characteristics and cancer-related interpretation bias has not been thoroughly examined and warrants attention.

### Current Study

The first aim of this study was to examine theoretically proposed relationships between interpretation bias, FCR, and somatic symptoms (Lee-Jones et al., 1997). We hypothesized that interpretation bias would be related to more FCR, and overall problematic somatic symptoms. Further, given the link between somatic symptoms and FCR, along with presumptive links between interpretation bias and both these constructs based on the cognitive formulation of FCR, the second goal of this study was to examine interpretation bias as a mediator of the association between somatic symptoms and FCR. We hypothesized that interpretation bias would mediate the relationship between somatic symptoms and FCR. A third exploratory aim was to examine associations between demographic and medical variables linked to FCR in relation to cancer-related interpretation bias to elucidate the role of these factors in cancer-related cognitions and to inform whether these variables fit in the cognitive formulation of FCR.

## Materials and Methods

### Participants

The current investigation utilized baseline data collected from October 2012 through November 2015 as a part of an RCT of a CBM intervention (Lichtenthal et al., 2017). Participants (*n* = 110) were English-speaking women (self-identified) ages 18 or older who were diagnosed with stages 0–III breast cancer, had no history of recurrence or metastases, and had completed active treatment for their breast cancer. Women were eligible if they scored at least a “3” on the Concerns About Recurrence Scale (CARS) Overall Fear Index (Vickberg, 2003), suggesting at least moderate FCR. Following Institutional Review Board approval, patients from a large urban cancer center were recruited through in-clinic approaches, mailed study invitations and telephone calls. Breast cancer survivors were screened for eligibility, and informed consent was obtained from those interested in participation. Participants received a total of $50 compensation for completion of the study. The current paper is a secondary examination of baseline characteristics reported by the trial participants.

### Measures

#### Interpretation Bias

The WSAP assessment in the current study consisted of cancer-specific stimuli given the goal of reducing cancer-related interpretation bias. The WSAP assessment required participants to determine whether benign or cancer-related words and sentences describing ambiguous situations were related. Each trial began with a fixation cross presented on a computer screen for 500 milliseconds (ms) to alert participants about the start. Subsequently, the fixation cross disappeared and a benign (e.g., “Sleep”) or threat (e.g., “Cancer”) word was presented on the screen for 500 ms. When the word disappeared, an ambiguous sentence appeared on the screen (e.g., “You have been tired lately”). Participants were then prompted to indicate whether they thought that the word and sentence were related (by pressing number “1” on the computer keyboard) or not related (by pressing number “3”). The next trial (i.e., fixation cross, a cancer-related word or benign word, an ambiguous sentence) appeared immediately after for a total of 118 trials. As done in prior studies using the WSAP, we used four separate interpretation bias metrics to assess the extent of interpretation bias toward cancer-related threat. We calculated (1) the percentage of cancer-related threat endorsement (i.e., “Rate of Threat Endorsement”), (2) the percentage of benign interpretation endorsement (i.e., “Rate of Benign Endorsement”), (3) the mean reaction time (RT) for threat endorsement, and (4) the mean RT for benign endorsement. Consistent with the WSAP literature (Beard and Amir, 2009), higher threat endorsement rates and lower benign endorsement rates were believed to indicate more interpretation bias. Similarly, faster RT for threat endorsement and slower RT for benign endorsement were theorized to indicate more interpretation bias.

#### Fear of Cancer Recurrence

Fear of cancer recurrence was measured using the CARS (Vickberg, 2003), a widely used, reliable and valid 30-item self-report instrument that that assesses the extent and nature of women’s FCR across five domains. Subscales include Overall Fear – assessing frequency/intensity of FCR using four questions (e.g., “How much time do you spend thinking about the possibility that your breast cancer could recur?”) with a response scale ranging from 1 (*I don’t think about it at all*) to 6 (*I think about it all the time*), Health Worries (11 items; e.g., “I worry that a recurrence of breast cancer would threaten my physical health.”), Womanhood Worries (seven items; e.g., “I worry that a recurrence of breast cancer would interfere with my sense of sexuality.”), Role Worries (six items; e.g., “I worry that a recurrence of breast cancer would keep me from fulfilling my responsibilities [in my job or at home.]”), and Death Worries (two items; e.g., “I worry that a recurrence of breast cancer would cause me to die”). Final scores were computed by averaging responses. We used the Overall Fear score in main analyses, and the remaining subscale scores in *post hoc* analyses.

#### Overall Somatic Symptoms

Overall somatic symptoms were assessed using eight questions from the Physical Well-Being subscale of the quality of life-cancer survivors measure (QOL-CS; Ferrell et al., 1995). QOL-CS is a 41-item valid and reliable instrument (Pearce et al., 2008) designed to assess somatic, psychological, social, and spiritual well-being in cancer survivors. The Physical Well-Being subscale assessed the extent to which quality of life was affected by the cancer experience across eight different somatic symptoms: fatigue, appetite changes, aches or pain, sleep changes, weight gain, vaginal dryness/menopausal symptoms, menstrual changes or fertility, and physical health. The response scale ranges from 0 (*no problem*) to 10 (severe problem) and was recoded so that a lower score would represent a stronger severity of the symptom (indicating lower quality of life) in any of the above-named domains. We used the *overall problematic somatic symptoms* score in main analyses, and specific symptoms scores in *post hoc* analyses.

## Data Analysis

To examine associations between cancer-related interpretation bias metrics, Overall Fear, and overall problematic somatic symptoms (aim 1), we calculated a series of Pearson’s correlations. Next, we explored cancer-related interpretation bias as a mediator of the link between somatic symptom score and the Overall Fear score (aim 2). The mediation model was identified based on significant associations between a predictor (i.e., overall problematic somatic symptoms score) and a mediator (i.e., an index of cancer-related interpretation bias), and an outcome (i.e., Overall Fear score), and a predictor and an outcome. To test mediation, we used the PROCESS macro for SPSS (Hayes, 2013), which calculates 95% bias-corrected confidence intervals of indirect effects using 1000 bootstrap samples. To test exploratory relationships between medical and demographic factors and cancer-related interpretation bias (exploratory aim 3), we used independent samples *t*-tests for associations between categorical and continuous variables and Pearson’s correlations for associations between continuous variables. To determine the strength of the associations tested for each of these three aims, we calculated effect sizes (i.e., correlation coefficient (*r*) for Pearson’s correlations, Cohen’s *d* for *t*-tests, and standardized *B*’s for mediation). To account for multiple comparisons, we used the Benjamini–Hochberg (Benjamini and Hochberg, 1995) procedure (*Q* = 0.05) to adjust *p*-values for all examined associations, including those examined in mediational models. Finally, we conducted *post hoc* analyses to examine relationships between the remaining subscales of the CARS scale, and specific symptoms of the QOL-CS scale, and all indices of cancer-related interpretation bias.

## Results

### Participant Characteristics

Participants were 55 years old on average (SD = 8.10), highly educated (51% had more than a college degree) and mostly White, non-Latinx (74%). A detailed description of the demographic characteristics of participants is included in the publication of the RCT findings (Lichtenthal et al., 2017).

### Associations Between Cancer-Related Interpretation Bias and Fear of Cancer Recurrence

Greater rates of threat endorsement were associated with higher Overall Fear [*r*(92) = 0.30, *p* = 0.003]. Associations between Overall Fear and threat endorsement RT, benign endorsement, and benign endorsement RT were non-significant. See Table 1 for more details. *Post hoc* analyses revealed that higher scores on Health Worries and Role Worries subscales were each associated with greater rates of threat endorsement (*p* < 0.01). No other indices of interpretation bias were related to FCR.

**TABLE 1:** Baseline correlations between cancer-related interpretation bias and psychological and somatic factors.

	**Rate of threat endorsement**	**Threat endorsement RT**	**Rate of benign endorsement**	**Benign endorsement RT**
**Fear of recurrence (CARS)**				
CARS Overall Fear	*r* = 0.30^**^	*r* = −0.16	*r* = 0.08	*r* = −0.08
**Physical well-being (QOL-CS)**				
Overall problematic somatic symptoms	*r* = −0.29^**^	*r* = 0.09	*r* = −0.17	*r* = 0.09

*Unadjusted findings are reported.*

*p < 0.05, **p < 0.01, ***p < 0.001.

### Associations Between Overall Problematic Somatic Symptoms and Cancer-Related Interpretation Bias

Greater rates of threat endorsement were associated with more overall problematic somatic symptoms [*r*(92) = −0.29, *p* = 0.005]. Associations between the remaining indices of bias and overall problematic symptoms were not significant (*p* > 0.05). See Table 1 for additional details. Investigating associations between somatic symptoms rated as problematic and cancer-related interpretation bias showed that ratings of fatigue (*p* < 0.01), sleep changes (*p* < 0.001), menstrual changes or fertility problems (*p* < 0.05), and poorer physical health (*p* < 0.05) were all associated with higher rates of threat endorsement. *Post hoc* analyses revealed that ratings of fatigue (*p* < 0.01), sleep changes (*p* < 0.001), menstrual changes or fertility problems (*p* < 0.05), and poorer physical health (*p* < 0.05) were all associated with higher rates of threat endorsement.

### Associations Between Medical and Demographic Factors and Cancer-Related Interpretation Bias

Longer benign endorsement RT was associated with greater time since cancer treatment completion [*r*(92) = 0.21, *p* = 0.041] and older age [*r*(92) = 0.29, *p* = 0.005]. We also identified higher rates of benign endorsement of [*t*(91) = −1.99, *p* = 0.049, *d* = 0.50] in participants who had at least one child. Interpretation bias was not related to other demographic or medical variables (*p*s > 0.05). The Benjamini–Hochberg adjustment resulted in non-significant findings (adjusted *p*s > 0.05) for the relationships between bias, time since treatment completion, and having at least one child. See Table 2 for more information.

**TABLE 2:** Baseline associations between cancer-related interpretation bias and medical and demographic characteristics.

	**Rate of threat endorsement (%)**			**Threat endorsement RT (ms)**			**Rate of benign endorsement (%)**			**Benign endorsement RT**		
**Variable (*n*)**	***M* (SD)^*a*^**	***t***	**Effect size^b,c^**	***M* (SD)^d^**	***t***	**Effect size^b,c^**	***M* (SD)^e^**	***t***	**Effect size^*b,c*^**	***M* (SD)^f^**	***t***	**Effect size^b,c^**
**Stage**												
0 and 1 (47)	51.21 (16.00)	*t*(92) = −1.24	*d* = 0.26	1529 (602)	*t*(92) = −0.17	*d* = 0.03	72.48 (12.26)	*t*(92) = −1.03	*d* = 0.21	1369 (477)	*t*(92) = −0.45	*d* = 0.09
2 and 3 (47)	56.07 (21.50)			1551 (671)			75.51 (16.01)			1416 (550)		
**Minoritized status**												
White, not Hispanic (68)	51.43 (19.98)	*t*(90) = −1.92	*d* = 0.46	1532 (627)	*t*(90) = 0.16	*d* = 0.04	72.89 (15.24)	*t*(90) = −1.09	*d* = 0.26	1404 (502)	*t*(90) = 0.61	*d* = 0.15
Other (24)	60.07 (15.38)			1508 (657)			76.62 (11.46)			1329 (555)		
**Children**												
No (26)	54.32 (23.92)	*t*(91) = 0.28	*d* = 0.05	1527 (676)	*t*(91) = −0.20	*d* = 0.05	69.22 (20.03)	*t*(91) = −1.99*	*d* = 0.50	1372 (535)	*t*(91) = −0.29	*d* = 0.07
Yes (67)	53.08 (16.91)			1557 (619)			75.71 (11.01)			1407 (508)		
Time since treatment	–	–	*r* = −0.07	–	–	*r* = 0.10	–	–	*r* = −0.05	–	*r* = 0.21*	–
Age	–	–	*r* = 00	–	–	*r* = 0.17	–	–	*r* = −0.10		*r* = 0.29**	–

Unadjusted findings are reported. *p < 0.05, **p < 0.01. ^a^Lower mean value represents less bias. ^b^*d* = Cohen’s *d*. ^c^*r* = Pearson’s correlation. ^d^Higher mean value represents less bias. ^e^Higher mean value represents less bias. ^f^Lower mean value represents less bias.

### Interpretation Bias as a Mediator

**FIGURE 1:**
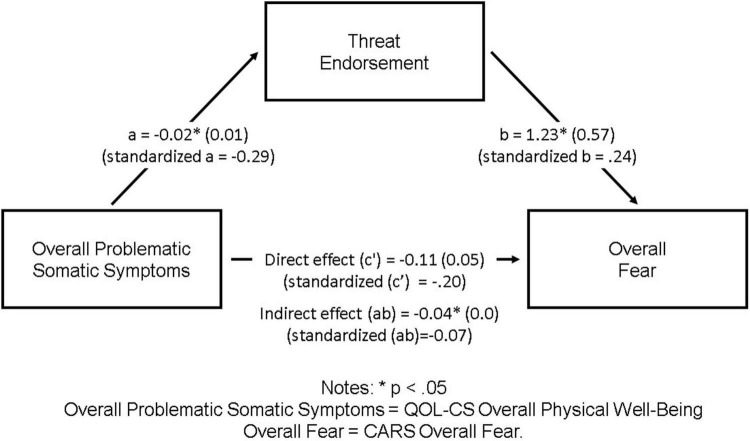
Threat endorsement as a mediator of the association between overall problematic somatic symptoms and Overall Fear. ^∗^*p* < 0.05. Overall problematic somatic symptoms, QOL-CS overall physical well-being; Overall Fear, CARS Overall Fear.

Based on significant associations (*p* < 0.05) between potential predictor, mediator, and outcome variables, threat endorsement was identified as a potential mediator of the association between Overall Fear and overall problematic somatic symptoms. Specifically, more problematic somatic symptoms were related to higher threat endorsement, and higher threat endorsement was subsequently related to higher Overall Fear. See Figure 1 for details.

## Discussion

The current study examined whether interpretation bias is associated with FCR, as prior research has suggested (Lee-Jones et al., 1997; Lichtenthal et al., 2017; Pradhan et al., 2021). We also examined somatic, demographic and medical correlates of cancer-related interpretation bias. In our sample of post-treatment breast cancer survivors, we found that the tendency to make threatening interpretations in the WSAP bias assessment was associated with Overall Fear, and overall problematic somatic symptoms. Mediation analyses further elucidated these links showing the mediating role of interpretation bias between overall problematic symptoms and increased FCR, which provides empirical validation of cognitive formulation of FCR (Lee-Jones et al., 1997).

Our examination of cancer-related interpretation bias and demographic and medical correlates showed that prior to *p*-value adjustments, age, time since treatment, and parent status were related to longer RT for benign but not threat interpretations. Breast cancer survivors who were older and those who were further out from their treatment took longer to react to benign interpretations. Although longer benign endorsement RTs are theorized to represent greater cancer-related interpretation bias, slowed processing speed can be partly a function of age-related changes in cognitive motor performance (Eckert et al., 2010) and/or cancer-related cognitive impairment (Pendergrass et al., 2018), rather than a marker of greater interpretation bias. It is also possible that those who were farther out from treatment may have developed strategies to initially avoid thinking about recurrence but that their cognitive biases can still be identified through the more implicit marker, slowed RT. Given that similar patterns did not emerge for slowed responses for threat, implies that the valence of the stimuli (i.e., neutral or threat) differentially impacts RT in people who are older or farther away from completing active treatment.

Having at least one child versus not having any, was associated with higher rates of benign endorsement. While research on parenthood and FCR is scarce, studies generally report that cancer survivors with children endorse higher FCR (Crist and Grunfeld, 2013). However, qualitative research suggests that parenthood in non-recurrent cancer can serve as a source of meaning and strength for continuing day-to-day activities (Arès et al., 2014). Thus, although having children may overall increase FCR worries, certain social profile characteristics may buffer against select negative consequences of FCR.

*Post hoc* analyses showed that greater threat endorsement was linked to health and role worries, which implies that health worries and worries about functional impairment in managing important responsibilities at work or home and social realm may drive Overall Fear, and are elicited, at least partly, by threat interpretations of ambiguous cues. These links warrant more attention and further emphasize the potential importance of intervening on interpretation bias. Threat endorsement was also linked to fatigue, sleep changes, menstrual changes or fertility problems, and ratings of poorer physical health. This elucidates which somatic symptoms may be most salient for somatic vigilance, cognitive catastrophizing, and FCR.

### Clinical Implications

This study contributes greater understanding of factors underlying cancer fears and is a critical step toward refining theoretical models of FCR. Given the links between interpretation bias and FCR, and that cancer-related bias can be reduced with intervention (Lichtenthal et al., 2017), these results suggest that negative cognitions may be an important intervention target in treating FCR. Mental health clinicians and health care providers should be made aware that those breast cancer survivors who tend to interpret ambiguous medical scenarios or somatic symptoms as a sign of cancer recurrence are also likely to have higher anxiety about cancer recurrence. It may be helpful to provide cancer survivors with psychoeducation about the link between cancer-related interpretation biases and FCR as well as concrete guidance about when symptoms are cause for concern. Cancer survivors walk a difficult line as they feel compelled to remain attuned to their bodies so that they can report concerning symptoms to their medical team while also wishing to interpret benign symptoms as such. Determining when to focus on their symptoms is a significant psychological challenge (Sharpe, 2019). However, given how hard it is to walk this line consciously, intervention approaches that operate on an implicit level of information processing, such as CBM, may hold promise (Lichtenthal et al., 2017).

### Study Limitations and Future Research Directions

This study was limited by the relatively small, homogenous sample, which consisted of primarily White, well-educated, ciswomen (i.e., persons whose gender identity matches their sex assigned at birth) pooled *via* convenience sampling from a large urban comprehensive cancer center. Although the eligibility specificity of our study sample (i.e., early-stage breast cancer survivors) allowed for identifying FCR triggers relevant to this group, these parameters limit the study’s generalizability to individuals with advanced metastatic disease, recurrent breast cancer, breast cancer survivors who identify as persons of color (Janz et al., 2011), those who do not identify as ciswomen (Kamen et al., 2015), those with less educational attainment (Koch et al., 2013), and those with different cancer types (Hall et al., 2018).

The study is also limited by the assessments of FCR and anxiety utilized. Research on FCR and its intersection with anxiety more broadly continues to evolve. Given individuals experiencing generalized anxiety disorder (GAD) are more likely to interpret ambiguous stimuli as threatening (Mathews et al., 1989), the extent to which generalized anxiety and related interpretation biases are driving our observed findings is unclear. Additional research is needed to tease apart these relationships and to further examine the relationships between medical and demographic characteristics and cancer-related interpretation bias.

Mediation was tested using a cross-sectional, small dataset rather than an adequately powered longitudinal dataset. This limits our ability to draw conclusions about temporal precedence and causality, although the observed results provide some preliminary data suggesting the relevance of interpretation bias in connecting physical symptoms with FCR and thus should be considered hypothesis-generating.

Overall, this study contributes to the literature on the role of cognitive biases, and specifically interpretation bias, on FCR. It also provided evidence that interpretation bias acts as a mediator of the relationship between internal symptoms and interpretation bias. Longitudinal investigations and studies that include external situations theorized to trigger emotional arousal (e.g., medical appointments) would provide for more robust understanding of the role of cues for interpretation bias, FCR and their relationships to profiles of cancer survivors.

## Data Availability Statement

The datasets presented in this article are not readily available because of ethical and privacy concerns. Requests to access the datasets should be directed to WL, lichtenw@mskcc.org.

## Ethics Statement

The studies involving human participants were reviewed and approved by the Memorial Sloan Kettering Cancer Center’s Institutional Review Board (IRB). The patients/participants provided their written informed consent to participate in this study.

## Author Contributions

MT and KR: conceptualization, project administration, database organization, methodology, statistical software, and formal analysis. GC: project coordination, database organization, methodology, and formal analysis. CB: conceptualization, investigation, supervision, data curation, methodology, and resources. CF and TC: formal analysis, statistical software, and resources. ESl: project administration, database organization, statistical software, methodology, and formal analysis. ESc: statistical consultation. WL: conceptualization, investigation, supervision, project administration, methodology, formal analysis, and funding acquisition. All authors contributed to manuscript writing, revision, read, and approved the submitted version.

## Conflict of Interest

The authors declare that the research was conducted in the absence of any commercial or financial relationships that could be construed as a potential conflict of interest.

## Publisher’s Note

All claims expressed in this article are solely those of the authors and do not necessarily represent those of their affiliated organizations, or those of the publisher, the editors and the reviewers. Any product that may be evaluated in this article, or claim that may be made by its manufacturer, is not guaranteed or endorsed by the publisher.
